# Development of a Microfluidic Paper-Based Analytical Device for Myeloperoxidase Detection in Periodontitis

**DOI:** 10.3390/dj13070321

**Published:** 2025-07-15

**Authors:** Juliane Caroline Leão, Thiago Mazzu, Vitor Leão, Paola Gomes Souza, Nathalya Maria Vilela Moura, Emanuel Carrilho, Mario Taba

**Affiliations:** 1 Department of Oral and Maxillofacial Surgery and Traumatology and Periodontology, School of Dentistry of Ribeirao Preto, University of Sao Paulo, Ribeirao Preto 14040-904, Sao Paulo, Brazil; dra.julianeleao@yahoo.com.br (J.C.L.); paolagomessouza@gmail.com (P.G.S.); nathalya_vilela@usp.br (N.M.V.M.); 2 Bioanalytics, Microfabrication and Separations Group, Chemistry Institute of Sao Carlos, University of Sao Paulo, Sao Carlos 13566-590, Sao Paulo, Brazil; thiagomazzu@gmail.com (T.M.); emanuel@iqsc.usp.br (E.C.); 3 Department of Genetics, School of Medicine of Ribeirao Preto, University of Sao Paulo, Ribeirao Preto 14049-900, Sao Paulo, Brazil; vitor.leao.vleao@gmail.com

**Keywords:** periodontal disease, myeloperoxidase, peroxidase, paper chromatographic test

## Abstract

**Objectives:** To develop a microfluidic paper-based analytical device (μPAD) that identifies myeloperoxidase (MPO) levels in the saliva of healthy patients and those with periodontal disease. **Materials and Methods:** A platform similar to a 96-well plate was printed on Watman^®^ chromatography paper to run the experimental analysis with unstimulated saliva samples were collected from two groups of patients: those with periodontal health (H, n = 15) and established periodontitis (PD, n = 15). Then, three types of chromophore substrates were pipetted into the wells of the prototype: (1) Guaiacol; (2) Guaiacol, 4,4 ′-diaminodifenilsulfon (DAB) and hydrogen peroxide in Tris-HCl buffer; and (3) 3,3′,5,5′-Tetramethylbenzidine (TMB), followed by saliva samples. The reaction images were analyzed by numbering according to the intensity scale. **Results:** The comparative results of the reactions using μPAD demonstrated that both the H and PD groups were compatible with each other without differences among the chromophore substrates (*p* > 0.05). However, the protocol with TMB showed a faster reaction and better color difference when comparing 15.62 ng/mL and 7.81 ng/mL of MPO in the plate embedded with Guaiacol; 1000 ng/mL and 62.5 ng/mL on the Guaiacol and DAB plate; and 62.5 ng/mL of TMB. The average detectable concentrations of MPO in saliva using TMB were H = 21.2 ± 10.4 ng/mL and PD = 28.9 ± 12.8 ng/mL (*p* = 0.08). **Conclusions:** The developed microfluidic paper-based analytical device has been tested for identifying the myeloperoxidase saliva levels of healthy patients and those with periodontal disease. This rapid test demonstrated its possible applicability mainly when associated with the TMB chromophore, but further studies are required with different biomarkers to explore this promising diagnostic platform.

## 1. Introduction

Periodontal disease is a chronic inflammatory polymicrobial disease induced by periodontopathogenic bacteria that has systemic implications [1]. These bacteria are considered to be the disease’s etiological agents because they present numerous virulence factors; however, most of the cell destruction, affecting the connective tissue and alveolar bone, occurs due to the host’s response [2]. Thereby, the inflammatory response induced by dysbiotic biofilm is the main cause of the disease, but it is the host’s innate susceptibility that determines the outcome [3,4,5,6]. Therefore, the treatment planning stage is critical and thorough, and a new complementary diagnostic tool, together with clinical and radiographic examination, can provide more accurate diagnostic methods, increasing the comprehension of periodontal diseases, optimizing treatment predictability and improving clinical management [7,8].

In this context, saliva is a secretion generated by the salivary glands that are of great importance in maintaining health. It is considered to be a mirror of oral and systemic health, and a source of clinically relevant information, as it contains specific biomarkers for physiological aspects [9,10,11]. For this reason, over the last few decades, it has become a diagnostic tool for identifying biomarkers [7,8,12]. Biomarkers play an important role in science and have begun to achieve importance in the diagnosis and early identification of periodontitis. Furthermore, they also facilitates treatment, in terms of prevention, early intervention, monitoring the results of therapy, contributing to oral and systemic health improvement, and patient referral [13,14,15].

When comparing a healthy periodontium versus a gingivitis/periodontitis scenario, several studies regarding salivary biomarkers demonstrate that there are differences regarding the concentrations of inflammatory markers such as TNF-α, IL-1β, IL-6, PCR, total MMP-8 (tMMP-8), active MMP-8 (aMMP-8), MMP-9, MMP-13, (MMP-8 being the only one capable of determining severe alveolar bone loss), albumin, osteoprotegerin, and myeloperoxidase (MPO), among others. Concerning key biomarkers such as MMP-8 and MPO, several studies, such as those developed by Aji et al. (2024) [16] and Thomas et al. (2024) [17], explain that there is a correlation between periodontal disease severity and those markers of salivary concentration since they are related to tissue destruction through the active response to an inflammatory stimulus. Therefore, throughout the years, science has been advancing in studies that use saliva as a tool in diagnosing periodontal diseases. They use biomarkers present in the saliva to identify and monitor the progression of these diseases [18,19,20].

Regarding aMMP-8, Aji et al. (2024) [16] demonstrated that this biomarker can be effectively used for evaluating treatment responses in periodontal diseases, as well as for diagnostics and disease screening. They also highlighted that, when compared to tMMP-8 and clinical indexes such as bleeding on probing (BOP), aMMP-8 is more accurate, effective, and superior in detecting both early and advanced periodontitis. The authors explained that tMMP-8 and aMMP-8 are not equivalent in periodontitis/peri-implantitis diagnostics, because tMMP-8 is less efficient in periodontal disease diagnostics and monitoring. Thomas et al. (2024) [17] also lay emphasis on how MMP-8 and MPO are key biomarkers associated with periodontal inflammation, but aMMP-8 is better associated with subclinical tissue destruction.

Myeloperoxidase (MPO) is an enzyme that enhances tissue damage in several pathologies. It is also an indicator of an increase in neutrophils and, consequently, tissue inflammation. Peroxidase is the class of enzymes to which MPO belongs, and it is responsible for catalyzing the oxidation of thiocyanate to hypocyanate in the presence of hydrogen peroxide. In other words, these are important biological antioxidants that fight bacteria and inactivate toxic substances. Higher levels of myeloperoxidase are linked to a worse periodontal condition, since MPO is associated with the oxidative stress pathways and it intervenes on host–microbial response modulation, enhancing its potential as a periodontal biomarker, as explained in the research developed by Thomas et al. (2024) [17]. Also, authors such as Aji (2024) [16] and Thomas et al. (2024) [17] have emphasized that MPO is important and efficient in detecting periodontitis since it can be a pro-oxidative activator of MMP-8 and can induce the production of reactive oxygen species, which can aggravate periodontal diseases. The conventional methods for MPO quantification and detection are usually high cost, require more knowledge and dominance over the procedure, and require long periods of time. Therefore, new ways of detecting MPO with better practicality are necessary [20,21,22,23,24,25].

Aji et al. (2024) [16] proved in their research that the concentration levels of tMMP-8, aMMP-8, MPO, PMN elastase, interleukin-6, and calprotectin, as well as clinical indexes such as the probing pocket depth, clinical attachment level, visible plaque index, and bleeding on probing significantly decreased after non-surgical periodontal treatment. Their results showed that the MPO, PMN elastase, and aMMP-8 catalytic activity assays were superior to the other tested biomarkers (tMMP-8, interleukin-6, and calprotectin). Therefore, those markers can be considered as an intervention target for periodontal treatment.

One of the diagnostic mechanisms that uses biomarkers in saliva is the use of microfluidic paper-based analytical devices (μPADs), which are based on wax printing on paper to create microfluidic devices that use capillary action to manipulate fluids for diagnostic assays. Some advantages of these devices are their small size and light weight, facilitating portability and requiring a small volume of the sample and reagent. Another advantage is their low cost, because they use low-cost materials and processing, which also makes them easy to use. They are particularly suitable for diagnostic applications with limited resources, easy to use, safe, and compatible with colorimetric assays [26,27,28,29].

Bhakta et al. (2014) [26] developed μPADs to identify and quantify nitrite levels in saliva. Nitrite is an antimicrobial agent produced from nitric oxide (NO) released by endothelial NO synthesis and, in saliva, it has been associated with periodontitis. Currently, there are several salivary tests available on the market for diagnosing and monitoring periodontal diseases based on biomarkers, DNA, and saliva’s buffering capacity, among others; these include the following: PerioSafe (measures MMP-8 levels in saliva, acting as an aid in the detection of active periodontal inflammation); OraRisk (analyses DNA for detection of periodontal pathogens); and Saliva-Check BUFFER (evaluates the buffering capacity of saliva) [27,30].

Therefore, this study aims to develop a microfluidic paper-based analytical device (μPAD) that identifies myeloperoxidase (MPO) levels in the saliva of healthy patients and those with periodontal disease.

## 2. Materials and Methods

After this study received approval by the Ethics Committee (CAAE n°: 27845414.8.1001.5419), thirty patients who met the following inclusion criteria were included: presence of at least 15 teeth (third molars were excluded); no periodontal treatment in the last 6 months; absence of systemic diseases; non-smoker; non-pregnant; no use of antibiotics, steroidal or non-steroidal anti-inflammatory drugs or immunosuppressants one month prior to the research; and aged over 35 years old.

Those patients underwent a complete periodontal examination determining 2 groups: the healthy group (H) and the periodontal disease group (PD). In both groups, the following parameters were evaluated:

- Age
- Number of teeth
- Probing pocket depth (PPD) in millimeters: evaluated using a periodontal probe. This is the distance from the gingival margin to the deepest area at which the probe can penetrate under light and constant force (25 N) until resistance is observed.
- Bleeding on probing (BOP). This is an index analyzed by recording bleeding sites after PPD examination.
- Clinical attachment level (CAL). This is the distance from the base of the periodontal pocket to a fixed point on the crown, such as the cemento-enamel junction (CEJ)
- Number of sites with a probing pocket depth between 4 and 6 mm
- Number of sites with a probing pocket depth greater than 7 mm.

The patients received a sterile falcon tube to collect unstimulated saliva (~3 mL) preserved in a −80 °C freezer [31].

- H (n = 15)—Patients with good periodontal health (absence of bleeding on probing—BOP < 10%; probing pocket depth <3 mm; absence of alveolar bone loss; absence of gingival edema or color change);
- PD (n = 15)—Patients diagnosed with periodontitis stage 3 grade B (loss of clinical attachment level >5 mm; maximum of 4 teeth lost due to periodontitis; probing pocket depth ≥6 mm; periodontal loss equivalent to plaque levels; less than 2 mm of bone loss/attachment in the last 5 years).

Recently, Valdivieso et al. (2025) [25] presented a novel approach for the electrochemical detection of MPO using printed screen graphene electrodes in the saliva of 30 subjects with periodontitis determined as a convenience sample. The authors highlighted the potential of the new method and MPO as a robust biomarker for periodontal disease.

To estimate the sample size for the clinical condition difference (PD and CAL), independent data were considered. Assuming that the standard deviation was similar in the two randomization groups, at about 30% of the mean value and a medium effect size using Cohen’s d with an α risk of 0.05, 15 subjects per group would guarantee 90% power to detect a minimum true difference of 0.8 points in the means.

**Figure 1:**
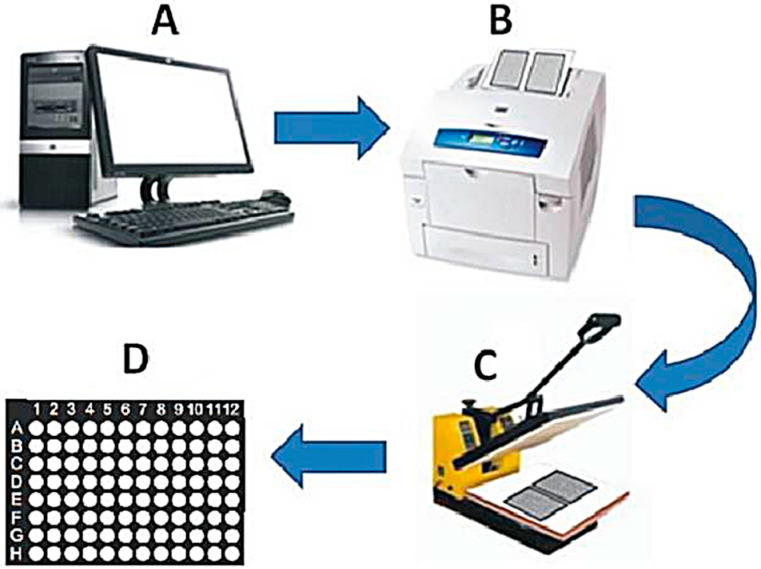
The µPAD manufacturing process using a wax printer. (**A**) Layout development; (**B**) wax printing; (**C**) heating the microplates at 150 °C for 2 min; (**D**) final microdevice; letters indicate rows and numbers indicate columns, like a regular 96-well plate.

Salivary fluid collected from both groups was used in the developed μPADs. The developed device contained in its structure a platform similar to a 96-well plate, with 8 mm diameter circles, arranged in eight rows of twelve circles overlapping a black rectangle; this was designed using the CorelDRAW^®^ Graphics Suite (X5 version) software. This platform was then printed on Whatman^®^ (VWR; Radnor, PA, USA) chromatography paper using the Xerox Phaser wax printer 8560 (Norwalk, CT, USA). Afterwards, the μPADs were heated in a press at 150 °C for 2 min, melting the wax that went through the thickness of the paper, delimiting the microzones where reactions occur. Since the wax has hydrophobic characteristics, it prevents liquids from leaking from the channels. The dimensions of the finished μPADs were 24 mm by 24 mm, with a 2 mm width for the main channel and 3 mm diameter for the testing zone [26] (Bhakta et al., 2014). After cooling to room temperature, the plates were suspended on a device so that the platform did not come into contact with the surface of the bench during the reaction (Figure 1).

Subsequently, a molarity curve was established for myeloperoxidase (MPO) (Myeloperoxidase from human leukocytes, ≥50 U/mg, Sigma-Aldrich, Saint Louis, MO, USA) and peroxidase (POx) (Peroxidase from horseradish type IV, 250–330 U/mg, Sigma-Aldrich), respectively, before plating samples from both groups onto the device. Dilution started from 1000 ng/mL, 500 ng/mL, 250 ng/mL, 125 ng/mL, 62.5 ng/mL, 31.25 ng/mL, 15.62 ng/mL, and 7.81 ng/mL. Hereafter, three types of chromophore substrates were prepared:

(1) Guaiacol (according to the manufacturer specifications—Sigma-Aldrich);
(2) The 176 mM Guaiacol, 3.47 mM DAB (4,4′-diaminodiphenylsulfon) (DAB) and 4 mM hydrogen peroxide in a 0.3 M Tris-HCl buffer (pH 7.5) [32];
(3) 3,3′,5,5′-Tetramethylbenzidine (TMB) [33].

So, 5 µL of each chromophore was pipetted onto the plate and allowed to dry for approximately 30 min, protected from light. Afterwards, 5 µL of each sample was pipetted in technical triplicate into each well, and one well contained milliQ water for the negative control.

The plates were photographed using a Kodak easyshare touch digital camera, from a distance of approximately 20 cm, without a flash, under the light intensity of the chapel. For the chromophores Guaiacol and DAB + Guaiacol, the photographs were taken after 4 min, for the chromophore TMB, due to the fact that the reaction was faster, after 1 min and after 2 min, 5 µL hydrochloric acid (0.01 M, Synth, Diadema, SP, Brazil) was used to stop the reaction, and new photographs were taken. All of this was carried out with the wells still being wet, due to the impossibility of scanning. After the plates dried (approximately 40 min), scanning was performed using the HP Scanjet 300 scanner. The images were analyzed with the Photoshop CS program, using the Magic Wand Tool, which was quantified by numbering according to the RGB scale, and these values were transformed into an increasing scale.

The data were grouped and presented as the mean and standard deviation after passing the normality test. The statistical differences were verified with the Prism statistical software (version 8) through ANOVA followed by a post-hoc Tukey’s test for chromophores, and Student’s *t*-test for H and DP group comparisons.

## 3. Results

Table 1 presents the periodontal parameters evaluated in the two groups studied: healthy group (n = 15) and periodontal disease group (n = 15). The mean age was 41 years in the healthy group and 48 years in the periodontal disease group, with no statistically significant difference (*p* = 0.0585). The mean number of teeth was significantly lower in the periodontal disease group (17.0 ± 4.35) compared to the healthy group (22.0 ± 3.57), with *p* = 0.0018. The mean PPD was significantly (*p* = 0.0001) higher in the periodontal disease group (2.11 ± 0.30 mm) compared to the healthy group (1.31 ± 0.05 mm). BOP was 28.0 ± 8.0% in the periodontal disease group and absent in the healthy group (*p* = 0.0001). The CAL was higher in the periodontal disease group (2.59 ± 0.54 mm) than in the healthy group (1.77 ± 0.56 mm), with statistical significance (*p* = 0.0003). The number of sites with a probing pocket depth of between 4 and 6 mm was 4.20 ± 5.09 in the periodontal disease group and absent in the healthy group (*p* = 0.0034). Similarly, sites with a probing pocket depth ≥7 mm were observed only in the periodontal disease group (1.00 ± 1.0), with statistical significance (*p* = 0.0043).

**Figure 2:**
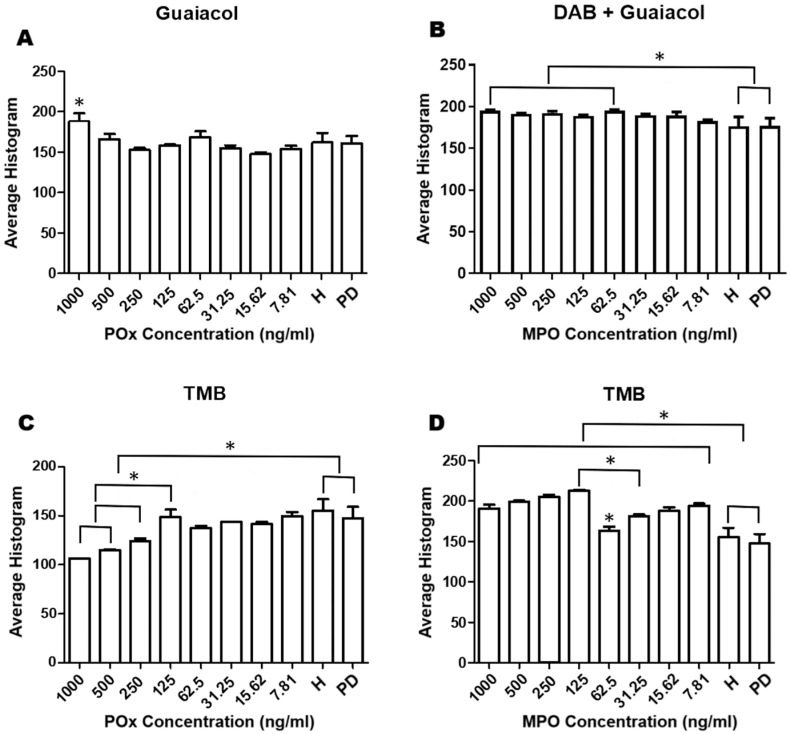
Data are presented as mean ± SD of enzyme concentrations with statistically significant values in each chromophore evaluated for the healthy (H) group and the group with periodontal disease (PD). (**A**) Peroxidase under Guaiacol; (**B**) myeloperoxidase under Guaiacol, DAB, and hydrogen peroxide in Tris-HCl buffer; (**C**) peroxidase under TMB; and (**D**) myeloperoxidase under TMB. Asterisks (*) indicate statistically significant difference (*p* ≤ 0.05).

Concerning the analysis on the developed platform between the evaluated chromophores, regarding Guaiacol, when the peroxidase curve (POx) was evaluated, the concentration of 1000 ng/mL showed a difference with all concentrations below 250 ng/mL (*p* ≤ 0.05). Regarding the analysis between the POx curve and the H and PD groups, there was a difference in the concentration of 1000 ng/mL (*p* ≤ 0.05) (Figure 2A). For the Guaiacol chromophore associated with DAB, it was shown that in the comparison between the myeloperoxidase (MPO) curve and the H and PD groups, they were different at concentrations of 1000 ng/mL (*p* ≤ 0.05) and 62.5 ng/mL (*p* ≤ 0.05) (Figure 2B).

The TMB chromophore, when evaluated, showed that in the POx curve, the concentration of 125 ng/mL was statistically different when compared to the concentrations of 1000 ng/mL and 500 ng/mL (*p* < 0.05) (Figure 2C). There was a difference between the H and PD groups when compared to the concentrations of < 125 ng/mL (*p* < 0.05) (Figure 2D). For the MPO curve, the concentration of 62.5 ng/mL presented a statistical difference with all groups, while the concentration of 125 ng/mL was also different from 31.25 ng/mL (*p* < 0.05). There was also a difference between the H and PD groups when compared to all MPO concentrations (*p* < 0.05) (Figure 2D).

**Figure 3:**
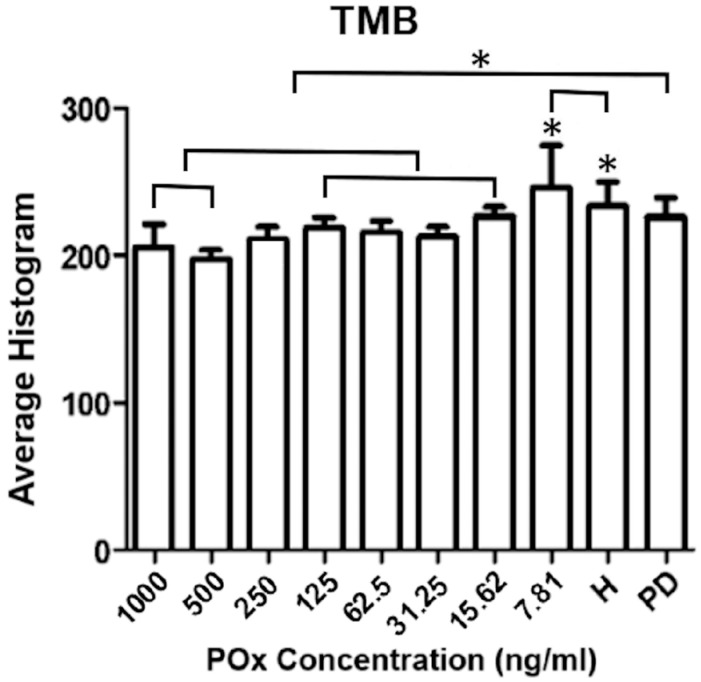
Data are presented as mean ± SD of POx concentrations and mean of Groups H and PD. µPAD under TMB chromophore photographed after 1 min of reaction. Asterisks (*) indicate statistically significant difference (*p* ≤ 0.05).

Based on the results described, the TMB chromophore showed the greatest statistically significant differences between the enzymes evaluated and between the sample groups. Therefore, TMB was selected for the scanning stage. In this stage, the results showed that, when evaluating the POx curve, the H group was equal to the concentration of 7.81 ng/mL and different from all other concentrations (*p* < 0.01). The PD group was statistically different from the concentrations: 1000 ng/mL, 500 ng/mL, 125 ng/mL, 15.62 ng/mL, and 7.81 ng/mL (*p* ≤ 0.01) (Figure 3).

**Figure 4:**
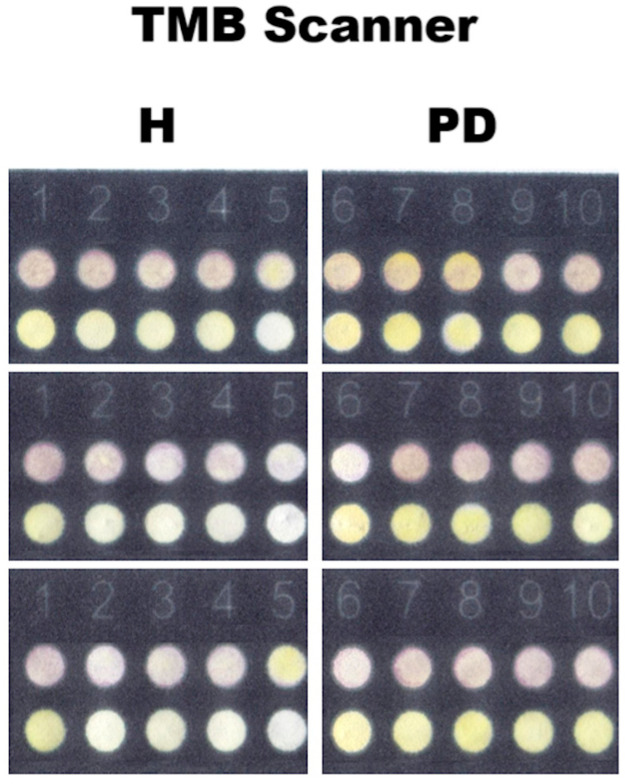
Scanned images of the plates with TMB + HCl chromophore. Lines representing POx concentrations; experiment carried out in biological duplicate and technical triplicate (1–5: healthy group (H) and 6–10: with periodontal disease group (PD).

In summary, the results showed that, both the group with periodontal disease and the healthy group, when compared to each other, showed similarity for all chromophores with regard to MPO concentrations. However, TMB was the most sensitive in detecting different levels for the POx and MPO curves in comparison to the others (Guaiacol and Guaiacol + DAB). This chromophore yielded superior results, exhibiting improved application simplicity, faster reaction kinetics, and enhanced color differentiation (Figure 4).

A post hoc power analysis (G*Power 3.1.9.7) for clinical status demonstrated a power of 1.00 for PPD and 0.98 for CAL. These high-power values confirm the significant differences observed between the healthy and diseased groups. Concerning the post hoc MPO analysis, a power = 0.42 is in agreement with the observed non-significant *p*-value between groups and corroborates the findings of limited specificity of this biomarker.

## 4. Discussion

The conventional clinical periodontal parameters are diagnostic strategies that provide information about the severity of periodontal disease, as in the results obtained in this study, which demonstrate the distinction between the H and PD groups. However, these parameters do not represent the activity of gingival inflammation; therefore, complementary alternatives that aim for greater precision in prevention and early intervention optimize treatment predictability and improve the clinical management of patients. So, the detection of salivary biomarkers associated with pathogenesis is of extreme importance [34,35,36].

In the present study, the objective was to develop a microfluidic paper-based analytical device (μPAD) that identifies myeloperoxidase (MPO) levels in the saliva of health patients and those with periodontal disease. Colorimetric assays in μPAD are a promising approach to detect biological processes, being able to detect the activity of proteases and providing accurate techniques in the diagnosis and monitoring of inflammation such as in periodontal diseases [37,38]. However, colorimetric assays have limitations with regard to sensitivity and specificity, mainly because the substrates are not specific, and contamination of biological samples with inhibitors can occur frequently [33].

Both the group with periodontal disease and the healthy group, when compared to each other, showed similarity for all chromophores with regard to MPO concentrations. This can be derived from the difficulty in stabilizing the reagent on the paper platform, which was also observed in a previous study [26]. When we compared the groups with the curves, the plate, after pipetting the stop, showed similarities with low concentrations of MPO, unlike the scanned plate, which showed similarity with high concentrations. This may be due to the platform lightening during drying. When pairing the groups with peroxidase, there was similarity between all of them with equal to or less than 125 ng/mL of peroxidase; demonstrating that peroxidase reacted in a constant way, like saliva when using TMB, or after the stop application, or after drying.

The data used were the average of the groups, so equivalence was determined; however, as there is a difference between individuals previously placed in the same group, we can consider that the biomarker is correlated to some specific concentration; and that other factors should determine the presence of MPO in saliva. Several individuals were classified as being sick or not, but the severity was not determined, which could have interfered with the results. Data relating to anamnesis may also have been neglected by the patient, due to the lack of a diagnosis of a systemic disease. MPO, being related to neutrophils, may be present more in gingivitis than in chronic disease, where other defense cells are also related. Serum MPO levels are susceptible to age, sex (being higher in women compared to men), smoking in men, and the use of oral contraceptives by women, in addition to other factors that need to be studied [39,40].

Regarding the results of the chromophores used when compared, it was observed that TMB was the most sensitive and therefore the best staining method for the POx and MPO curves. Compared to the others (Guaiacol and Guaiacol + DAB), this chromophore demonstrated more prominent results, accompanied by a greater ease of using the technique, rapid reaction, and better color difference. This agrees with the results of other studies present in the literature that used this chromophore in biosensors for protein identification in biological fluids and noted its excellent contribution and applicability in oxidation reactions, generating the characteristic blue color [41,42].

However, TMB is a light-sensitive substance that undergoes oxidation, enabling short-term analysis. An alternative approach involved using hydrochloric acid as a stop solution, but this acid compromises the technique’s effectiveness. Considering this, the most relevant results would come from the plates with TMB before the application of the stop. This is proven by studies that aim to catalyze the oxidation reaction proposed by TMB, that is, the acceleration of hydrogen donation for the reduction of hydrogen peroxide proposed by this chromophore has greater advantages in chemical terms despite the technical limitations [43,44].

Regarding the device, the determination of the MPO curve had low specificity; however the best parameters were blocked when initially photographed, despite the low sensitivity of the platform. Peroxidase showed differences between concentrations, but they were not completely understood. This leads us to believe that the method of evaluating and capturing the image may have compromised the data. The evaluation of color density by an optical device containing a light-emitting diode (LED) at a wavelength of 460 nm and a photodetector, as used by Sakamoto et al. (2008) [33], could be useful to see if it has a better image quality. The type of data collection in the Photoshop program should also be further studied.

Despite the limitations of the paper-based device, there are a multitude of possibilities for the future use of oral fluids in biotechnology and healthcare applications, particularly in the diagnostics field. The future is bright for fast, easy diagnostic methods that provide enhanced assessment that can guide and transform personalized therapies for dental patients that are more individualized and targeted toward oral health [45]. There is a need for much additional research in this area before the true value of saliva as a diagnostic fluid can be determined [46]. This is because major challenges for diagnosing periodontal disease using saliva surround the science: current tests are based on a small number of potential biomarkers, and tests are heavily based on inflammatory cytokines that may or may not be disease specific. For salivary diagnosis, for periodontal diseases to be clinically relevant, appropriate bioinformatics have to enhance the discovery of specific biomarkers, or the development of an oral diagnosis based on a combination of markers [47].

Previous articles have already determined that MPO activity in the saliva may reflect the severity of periodontal disease [48,49]. However, this study is the first to focus on evaluating the correlation between periodontal disease and salivary diagnosis by paper-based devices using MPO as a molecular biomarker.

MPO’s specificity is not absolute, and its non-specific activity involves different pathological processes related to damage to tissues, defense against pathogens, and general inflammation, limiting its application when used alone, especially when dealing with subclinical gingival inflammation that is part of a normal host response.

Several authors, such as Brazaca et al. (2024) [27], Alahmad, Sahragard and Varanusupakul (2021) [50], and Hamedpour et al. (2020) [51] explain that, although the μPAD has demonstrated huge potential regarding aspects such as environmental monitoring, food safety assurance, and clinical diagnostics, in terms of being considered as a possible rapid-test platform, some issues are faced, especially regarding the following: (I) digital image processing (qualitative colorimetric tests are subjective and the interpretation of results by users can lead to inaccuracies); (II) the sensitivity and specificity to detect analytes (comparable to laboratory-based gold standards) especially for detecting low concentrations of biomarkers in complex biological samples; (III) the stability and storage capacity to ensure the long-term stability of reagents immobilized on paper, particularly under varying environmental conditions (temperature, humidity), which is crucial for practical applications; and (IV) sample challenges, such as viscosity, collection time, collection type (as in saliva; Bellagambi et al., 2020 [52] state that it can be collected through swab-based methods, spitting, dried saliva spots, passive drooling, and draining). All of those factors can interfere with the final results; hence, the color distribution and analysis.

Therefore, μPADs face several challenges that need to be overcome and require further developments for broader commercialization and clinical adoption. For these challenges, strict control over environmental humidity during reagent preparation and storage as well as the rapid execution of assays after reagent exposure would help. Alahmad, Sahragard and Varanusupakul (2021) [50] expand on this line of reasoning by agreeing that sensitivity is one of the challenges faced by μPADs and they suggest “preconcentration techniques” as a possible solution to enhance the sensitivity. Also, exploring different biomarkers and developing reproducible algorithms using image processing software (e.g., ImageJ or Python libraries like OpenCV) for the standardized extraction of meaningful quantitative data with background subtraction would be interesting in order to move to a more robust and sound method aligned with the World Health Organization’s “ASSURED” [53] criteria for ideal point-of-care tests: being Affordable, Sensitive, Specific, User-friendly, Rapid and Robust, Equipment-free, and Deliverable to end-users.

Hamedpour et al. (2020) [51] corroborate those affirmations, as they explain in their study that a wide range of image analysis methods (Raspberry Pi, Arduino, ImageJ plugins, smartphone apps, and text-displaying devices) have been studied to enhance assay outcomes and they concluded that, considering software limitations and that readout results may vary between different people, a scanner integrated with a MATLAB-based algorithm (version R2020a) may be a better choice. Brazaca et al. (2024) [27] complement this by suggesting that the color distribution problem can be fixed through piling papers with different absorptions. They explained that, after the solution deposition, the paper placed on top (faster absorption) hydrates faster, distributing the solution with uniformity to the bottom paper (slower absorption), which avoids movements of rehydrated reagents, enhancing the test’s sensitivity.

## 5. Conclusions

The developed microfluidic paper-based analytical device demonstrated potential in identifying myeloperoxidase levels in saliva samples, with a limited discriminatory capacity to distinguish between healthy patients and those with periodontal disease. Therefore, this rapid test demonstrated its applicability mainly when associated with the TMB chromophore, but it requires further development and studies to explore this promising portable, cost-effective platform.

## Figures and Tables

**Table 1:** Values of periodontal parameters evaluators in both sample groups (H group and PD group, n = 15). Age, number of teeth, probing pocket depth in millimeters (PPD), bleeding on probing (BOP), clinical attachment level (CAL), number of sites with probing pocket depth between 4 and 6 mm, and number of sites with probing pocket depth greater than 7 mm were evaluated. The data are presented as mean and standard deviation and levels not connected by same letter indicating statistically significant differences (*t*-test, *p* ≤ 0.05).

Parameters	Health Group (n = 15)	Periodontal Disease Group (n = 15)	*p*-Value
Age	41.0 ± 9.61 ^a^	48.0 ± 9.83 ^a^	0.0585
n. teeth	22.0 ± 3.57 ^b^	17.0 ± 4.35 ^a^	0.0018
PPD (mm)	1.31 ± 0.05 ^b^	2.11 ± 0.30 ^a^	0.0001
BOP (%)	0.00 ^b^	28.0 ± 8.0 ^a^	0.0001
CAL (mm)	1.77 ± 0.56 ^b^	2.59 ± 0.54 ^a^	0.0003
PPD 4–6 (n)	0.00 ^b^	4.20 ± 5.09 ^a^	0.0034
PPD = 7 (n)	0.00 ^b^	1.00 ± 1.0 ^a^	0.0043

## Data Availability

The original contributions presented in this study are included in the article. Further inquiries can be directed to the corresponding author.

## Abbreviations

The following abbreviations are used in this manuscript:

μPAD	Microfluidic paper-based analytical device
MPO	Myeloperoxidase
H	Healthy
PD	Periodontal disease
DAB	Diaminodifenilsulfon
TMB	Tetramethylbenzidine
TNF-α	Tumor necrosis factor alpha
IL	Interleukin
MMP	Metalloproteinase
NO	Nitric oxide
DNA	Deoxyribonucleic acid
PPD	Probing pocket depth
BOP	Bleeding on probing
CAL	Clinical attachment level
POx	Peroxidase curve
